# Altered Intrinsic Properties and Inhibitory Connectivity in Aged Parvalbumin-Expressing Dorsal Horn Neurons

**DOI:** 10.3389/fncir.2022.834173

**Published:** 2022-07-08

**Authors:** Mark A. Gradwell, Kelly M. Smith, Christopher V. Dayas, Douglas W. Smith, David I. Hughes, Robert J. Callister, Brett A. Graham

**Affiliations:** ^1^Rutgers, The State University of New Jersey, New Brunswick, NJ, United States; ^2^Centre for Neuroscience, Science Tower, University of Pittsburgh, Pittsburgh, PA, United States; ^3^School of Biomedical Sciences and Pharmacy, Faculty of Health and Medicine, University of Newcastle, Callaghan, NSW, Australia; ^4^Brain Neuromodulation Research Program, Hunter Medical Research Institute, New Lambton Heights, NSW, Australia; ^5^Institute of Neuroscience Psychology, College of Medical, Veterinary and Life Sciences, University of Glasgow, Glasgow, United Kingdom

**Keywords:** spinal cord, mouse, aging, interneuron, pain

## Abstract

The incidence of pain symptoms such as allodynia are known to increase with age. Parvalbumin expressing interneurons (PVINs) within the dorsal horn (DH) of the spinal cord play an important role in allodynia whereby their inhibitory connections prevent innocuous touch information from exciting nociceptive pathways. Here we ask whether the functional properties of PVINs are altered by aging, comparing their functional properties in adult (3–7 month) and aged mice (23–28 month). Patch clamp recordings were made from PVINs in laminae IIi-III of parasagittal spinal cord slices. The intrinsic excitability of PVINs changed with age. Specifically, AP discharge shifted from initial bursting to tonic firing, and firing duration during current injection increased. The nature of excitatory synaptic input to PVINs also changed with age with larger but less frequent spontaneous excitatory currents occurring in aged mice, however, the net effect of these differences produced a similar level of overall excitatory drive. Inhibitory drive was also remarkably similar in adult and aged PVINs. Photostimulation of ChR2 expressing PVINs was used to study inhibitory connections between PVINs and unidentified DH neurons and other PVINs. Based on latency and jitter, monosynaptic PVIN to unidentified-cell and PVIN-PVIN connections were compared in adult and aged mice, showing that PVIN to unidentified-cell connection strength increased with age. Fitting single or double exponentials to the decay phase of IPSCs showed there was also a shift from mixed (glycinergic and GABAergic) to GABAergic inhibitory transmission in aged animals. Overall, our data suggest the properties of PVIN neurons in aged animals enhance their output in spinal circuits in a manner that would blunt allodynia and help maintain normal sensory experience during aging.

## Introduction

Pain severely impacts quality of life and its severity increases with age (Kovacs et al., 2005; Henderson et al., 2013; Patel et al., 2013). This is manifest in aged clinical populations as increased pain perception during repetitive noxious stimulation and thermal pain tests, increased responses to capsaicin, and decreased responses to endogenous analgesics (Zheng et al., 2000; Edwards and Fillingim, 2001; Edwards et al., 2003). These data suggest age-related changes occur somewhere in pathways involved in the detection and/or processing of sensory signals that promote the pain experience.

There are several sites in the pain pathway that could contribute to increases in age-related pain. Pre-clinical work suggests these changes do not occur in the periphery as the properties of “pain transmitting” peripheral sensory neurons are not markedly altered during aging (Sato et al., 1985; Bergman et al., 1999). This points to central changes being responsible for age-related increases in pain. As the spinal cord dorsal horn (DH) is the first central location where peripheral signals are modified within local pain circuits (Todd, 2010), a few pre-clinical studies have searched for changes that would cause dysfunction in normal pain processing mechanisms. For example, Iwata et al. (2002) showed aged (29–34 month old) DH neurons in rat exhibited increased spontaneous activity, enhanced responses to nociceptive stimuli, altered receptive field properties and reduced descending inhibition. Several important functional properties also change in DH neurons in aged mice (28–32 month old) including intrinsic excitability, excitatory drive, and a shift toward GABA-dominant inhibition (Mayhew et al., 2019). Together, these observations suggest the properties of DH neurons are modified with age and the aged DH essentially exists in a pro-nociceptive or hyperexcitable state.

The consequences of the changes listed above on the output of DH circuits are difficult to interpret without precise knowledge of the type of neuron being investigated (Graham et al., 2007). This is important as any age-related disruption in the properties of a specific type of DH neuron could impact pain, itch, thermal or tactile signaling in different ways. Likewise, the phenotype of the neuron-type (excitatory or inhibitory) exhibiting altered properties will also be critical. Broadly speaking, increased excitability at inhibitory connections would promote an antinociceptive state, whereas enhanced excitability at excitatory connections would be pronociceptive.

Parvalbumin expressing interneurons (PVINs) are important for pain processing in the DH (Petitjean et al., 2015). For example, genetic silencing of PVINs enhances activation of nociceptive circuits following innocuous sensory input and can produce tactile allodynia. Conversely, activation of PVINs reduces allodynia in neuropathic pain models. Several underlying circuits have been linked to these observations. For example, PVIN-mediated inhibitory connections provide presynaptic inhibition to the central terminals of myelinated primary afferents in the DH, as well as providing postsynaptic input to a diverse range of DH neurons including vertical cells, PKC gamma-expressing cells, and projection neurons (Petitjean et al., 2015; Boyle et al., 2019; Gradwell et al., 2021). Because of their well-established role in a defined pain circuit, we have therefore examined the impact of aging on the intrinsic excitability of PVINs, their excitatory and inhibitory inputs, and the inhibitory connections they make with neurons in the DH.

## Materials and Methods

### Animals and Tissue Preparation

All procedures were approved by the Animal Care and Ethics Committee at the University of Newcastle. Electrophysiological studies were performed on transgenic mice (both sexes) expressing the fusion protein channelrhodopsin2-eYFP (ChR2-YFP) in parvalbumin positive interneurons (PVINs). Animals were generated by breeding two Jackson Laboratories (JAX) supplied mouse lines: the Cre-dependent ChR2/YFP expressing mouse line Ai32[RCL-ChR2 (H134R)/EYFP] (JAX Stock # 012569); and the PV-Cre knockin mouse line PV*^cre^* (JAX Stock # 08069). We have previously shown this line restricts ChR2 expression to PV expressing interneurons (Gradwell et al., 2021). Animals were housed up to six per cage in a temperature and humidity-controlled environment and kept on a 12 h light/dark cycle with *ad libitum* access to food and water. Our “adult” PV*^cre^*;Ai32 mice were aged to 3–7 months (*n* = 56; 23 male, 33 female), whereas “aged” PV*^cre^*;Ai32 mice were 23–28 months old (*n* = 15; 12 male, 3 female). Data for each age group was first separated by sex and compared to assess for sex-specific properties in either population. No differences were detected, so data from both sexes were pooled in adult vs. aged comparisons. In addition, precisely equating mouse and human life stages is somewhat contentious, however, the ages of the mice we used satisfy accepted age timepoints where mouse age corresponds to adult and aged humans (Flurkey et al., 2007; Dutta and Sengupta, 2016).

This study used the same dissection and slice preparation techniques described previously (Gradwell et al., 2021). Briefly, mice were deeply anesthetized with ketamine (100 mg/kg i.p) and decapitated. Using a ventral approach, the lumbar enlargement of the spinal cord was rapidly removed, oriented to cut sagittal slices and glued to the stage of a vibrating microtome (Leica VT-1000S, Heidelberg, Germany). Parasagittal slices (200 μm thick) were cut in a bath of ice-cold sucrose-substituted ACSF that was continually bubbled with Carbanox (95% O_2_ and 5% CO_2_) to achieve a pH of 7.3–7.4. The ACSF contained (in mM): 250 sucrose, 25 NaHCO_3_, 10 glucose, 2.5 KCl, 1 NaH_2_PO_4_, 1 MgCl_2_, and 2.5 CaCl_2_. All slices were stored in an interface chamber containing ACSF (118 mM NaCl substituted for sucrose), bubbled with Carbanox, and maintained at room temperature (21–24^°^C). Slices were allowed to equilibrate for 1 h prior to recording.

### Electrophysiology

After equilibration, slices were transferred to a recording chamber and continually superfused with ACSF (chamber volume 0.4 ml; exchange rate 4–6 bath volumes/min). Recordings were obtained at room temperature (21–24^°^C). Neurons were visualized *via* a Nikon FN-PT microscope equipped with near-infrared differential interference contrast optics and camera (Jenoptik ProgRes MF cool, Jena, Germany). Recordings were made within the clearly discernible YFP-expressing plexus of PVINs in laminae IIi-III (Hughes et al., 2012). Our recordings sampled PVINs, identified by YFP expression and ChR2-mediated photocurrents, as well as unidentified neurons that were YFP negative and did not exhibit photocurrents. YFP fluorescence and photostimulation were achieved using a FITC filter set (488-nm excitation, 508-nm emission filters) with illumination delivered by a CoolLED pE excitation system. Patch pipettes (4–8 MΩ) were filled with either a potassium gluconate-based internal solution containing (in mM): 135 C_6_H_11_KO_7_, 8 NaCl, 10 HEPES, 0.1 EGTA, 2 Mg_2_ATP, 0.3 NaGTP, pH 7.3 (with KOH) when assessing AP discharge or excitatory synaptic transmission (internal solution osmolarity adjusted to 290 mOsm); or a cesium chloride-based internal solution containing (in mM): 130 CsCl, 10 Hepes, 10 EGTA, 1 MgCl_2_, 2 Na_2_ATP, and 0.3 NaGTP (pH adjusted to 7.35 with 1 M CsOH) when assessing inhibitory synaptic transmission. Liquid junction potential corrections were not applied, though these values were calculated as 14.7 mV (potassium gluconate) and 10 mV (cesium chloride), at 22^°^C. In optically evoked postsynaptic current (oPSC) recordings from PVINs, QX-314 was added to the internal solution to block fast activating sodium channels and avoid contamination of recordings with unclamped spikes.

All whole-cell recordings were first established in voltage-clamp (holding potential -70 mV). Data were amplified using a Multiclamp 700B amplifier (Molecular Devices, Sunnyvale, CA, United States) digitized online (sampled at 10–20 kHz and filtered at 5–10 kHz) *via* an ITC-18 computer interface (Instrutech, Long Island, NY, United States), acquired and stored using Axograph X software (Axograph X, Sydney, Australia). After obtaining the whole-cell recording configuration, series resistance, input resistance and membrane capacitance were calculated based on the response to a hyperpolarising voltage step (5 mV, 10 ms duration, holding potential of -70 mV). Spontaneous excitatory postsynaptic currents (sEPSCs) were obtained using a potassium gluconate-based internal solution (holding potential -70 mV). The recording mode was then switched to current clamp for assessment of AP discharge (membrane potential set at -60 mV by applying bias current). AP discharge was studied by injecting a series of depolarizing step-currents (800 ms duration, 20 pA increments, delivered every 8 s) into the recorded neuron. During this protocol sustained baseline voltage deflections (i.e., in parts of the voltage trace not containing APs) were limited to a membrane potential of -20 mV to prevent cell damage. Spontaneous inhibitory postsynaptic currents (sIPSCs) were recorded using a cesium chloride-based internal solution (holding potential -70 mV). Firing properties could not be assessed in these cells as cesium blocks AP discharge (Camp et al., 2006).

The properties of the inhibitory synaptic connections between PVINs and other DH neurons (i.e., unidentified neurons and PVINs), were assessed using an optogenetic approach. Whole field photostimulation was applied at suprathreshold intensity (16 mW, duration 1 ms, every 12 s) unless otherwise stated. This ensured generation of action potential discharge in PV^+^-ChR2/YFP neurons and allowed us to study postsynaptic currents in DH neurons. When connections were present, recordings in unidentified neurons exhibited a light-evoked synaptic current. Responses in PVINs included an immediate photocurrent at the onset of photostimulation, followed by a short latency synaptic current.

### Data Analysis

All data were analyzed offline using Axograph X software (Axograph X, Sydney, Australia). Only neurons with a series resistance <30 MOhms (filtered at 5 KHz) were retained for analysis (24.7 ± 1.1 vs. 24.2 ± 1.2 MΩ for adult and aged recordings, respectively; *p* = 0.812). AP discharge was classified according to previously published criteria (Graham et al., 2004, 2007). In agreement with this work, PVINs expressed one of two types of AP discharge: tonic firing, which is characterized by persistent AP discharge for the duration of the depolarising current step; or initial bursting where AP discharge is limited to the beginning of the depolarising step. Several AP properties were also examined. AP’s elicited by step-current injections were captured using a derivative threshold method (dV/dt > 15 V/s) with the inflection point during spike initiation defined as AP threshold. AP amplitude was defined as the difference between AP threshold and the spike’s maximum positive peak. AP half-width was measured at 50% of AP height. AP afterhyperpolarization (AHP) amplitude was taken as the difference between AP threshold and the maximum negative peak after the spike. Rheobase current was defined as the smallest current-step that would elicit at least one spike. AP latency was measured as the time between current step onset and when membrane voltage reached AP threshold. Instantaneous frequency was calculated from the reciprocal of the interspike interval in successive APs and spike frequency adaptation was determined as a ratio of instantaneous frequency for first and last APs in response to a single current step injection. Similarly, AP amplitude adaptation was determined as the ratio of first and last AP amplitude measurements. To compare AP discharge in adult and aged PVINs neuron-to-neuron variability in rheobase current was accounted for by normalizing responses to multiple current steps. Taking rheobase responses from all neurons as the first data point and then reporting subsequent responses in 20 pA increments above rheobase achieved this normalization.

Analysis of sEPSCs and sIPSCs was completed using a sliding template method (semi-automated procedure within Axograph software package). All captured events were inspected individually and excluded from analysis if they contained overlapping currents or had unstable baselines before the rise or during the decay phase of the current. The peak amplitude and rise time (calculated over 10–90% of peak amplitude) were measured for all accepted events and instantaneous frequency was calculated as the reciprocal of inter-event interval for successive events. Analysis of decay time constant (calculated over 20–80% of the decay phase) was performed on averaged sEPSCs and sIPSCs, generated by aligning the rising phase of all accepted events in a recording. Averaged sEPSCs and sIPSCs were also used to calculate synaptic charge, defined as the area under the trace and reported in coulombs; and sEPSC and sIPSC drive was calculated by multiplying the average synaptic charge by sEPSC or sIPSC frequency for individual recordings, reported in amperes. At least 20 events were captured for all measurements.

For optogenetic stimulation experiments, postsynaptic inhibitory currents evoked by PVIN photostimulation were assessed in two ways. When this analysis was undertaken in a PVIN, where the response comprised an immediate photocurrent and later synaptic response, the baseline current was set to zero immediately prior to the optically evoked synaptic current (onset of second peak). The amplitude of the photostimulation-evoked synaptic input was measured from this level. For PVIN-unidentified cell connections synaptic responses were measured from baseline just prior to photostimulation. The peak amplitude of responses was calculated from the average of 10 successive trials. Response latency for all recordings was measured as the time between the onset of photostimulation and the onset of the synaptic response. The goodness of fit for single and double exponential fitting was assessed using sum of squares error (SSE). The ratio of the SSE for the double vs. single exponential fit was used to identify recordings where the double exponential produced a substantially fit improvement. As fitting a double exponential will always improve fitting to some extent (Callister and Walmsley, 1996) we set a “fit improvement” threshold of >2 to identify oIPSCs that had two clear decay components.

### Statistical Analysis

Statistical analysis was carried out using SPSS v10 (SPSS Inc. Chicago, IL, United States). Variables were compared using independent Student *t*-tests otherwise indicated in text. For comparison of discharge phenotype and connectivity Pearson Chi-Square test was used as stated in the text. Statistical significance was set at *p* < 0.05. All values are presented as means ± SEM.

## Results

### Parvalbumin Expressing Interneurons Intrinsic Excitability

**FIGURE 1:**
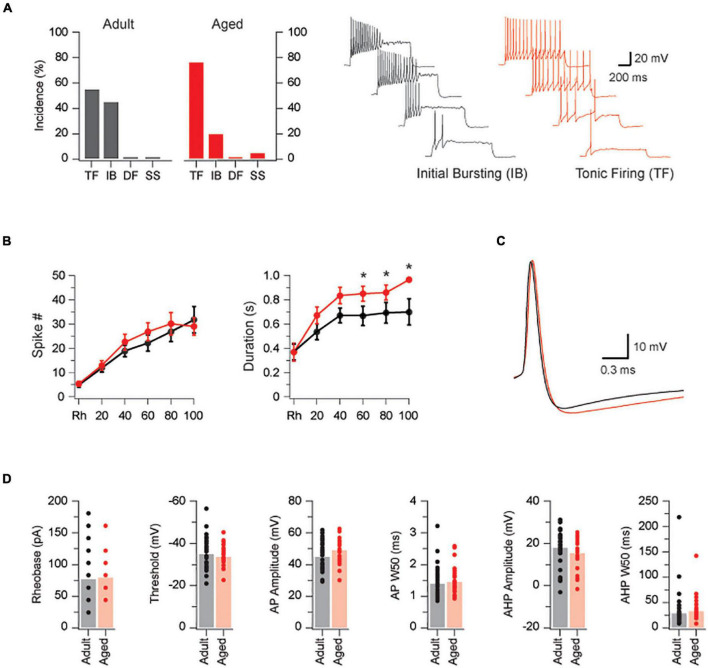
PVINs are more excitable in aged mice. **(A)** Bar graphs compare the incidence of AP discharge patterns in adult (black bars) and aged PVINs (red bars). TF and IB discharge dominate at both ages but there is a shift from IB to TF discharge in aged mice. Traces show the Initial Bursting and Tonic Firing AP discharge patterns in PVINs during depolarizing current step injections (20 pA step increments, 1 s duration). **(B)** Current-frequency and current-discharge duration relationships in adult (black dots) and aged (red dots) mice for five depolarizing current steps above rheobase. Note, AP discharge duration is enhanced in the aged population. **(C)** Overlaid traces of representative AP’s from adult (black) and aged (red) PVINs. **(D)** Group data comparing AP properties in adult (black) and aged (red) PVINs. None of the six AP properties (rheobase, threshold, AP amplitude, AP halfwidth – APW50, AHP amplitude, and AHP half-width – AHPW50) differed in adult and aged PVINs. *p < 0.05.

We first assessed the excitability of PVINs *via* targeted patch clamp recording from PVINs in adult (*n* = 33) and aged mice (*n* = 23). All recordings were restricted to PVINs located within the dense PVIN plexus in laminae IIi-III. Passive membrane properties of PVINs were similar in adult and aged animals (input resistance = 225 ± 22 vs. 185 ± 22 MΩ, *p* = 0.22; capacitance 10.9 ± 0.6 vs. 11.0 ± 0.6 pF, *p* = 0.90). The active properties of PVINs are summarized in Figure 1. AP discharge was examined by injecting depolarizing current-steps into PVINs (Figure 1A, Right traces). Using previously established criteria, responses were classified as tonic firing (TF), initial bursting (IB), delayed firing (DF), or single spiking (SS; Grudt and Perl, 2002; Graham et al., 2007). As with our previous studies on PVINs (Hughes et al., 2012), TF and IB responses dominated in both adult and aged neurons. TF and IB responses were equally represented in adult mice whereas TF was more dominant in aged animals [TF: 55% (18/33); IB 45% (15/33) vs. TF: 76% (16/21); IB 20% (4/21); SS 5% (1/21), *p* = 0.08 by Chi-Square test]. Spike frequency vs. current plots were similar in both groups (Figure 1B, Left plot), however, AP discharge duration was increased in aged animals for current injections greater than rheobase + 60 pA (*p* = 0.005, Figure 1B, Right plot). The increased duration of AP discharge coupled with the shift from IB to TF discharge in aged mice (plots in Figure 1A) would increase overall levels of excitability in aged PVINs.

The properties of individual APs were measured on rheobase APs in both age groups (Figure 1C and plots in Figure 1D). Rheobase current did not differ between the two age groups (77 ± 7 vs. 79 ± 8 pA, *p* = 0.82 Mann–Whitney *U* test). There were also no differences in AP threshold (-34.9 ± 1.3 vs. -33.6 ± 1.1 mV, *p* = 0.47), AP amplitude (44.8 ± 1.6 vs. 49.0 ± 1.9 mV, *p* = 0.10), AP half-width (1.4 ± 0.1 vs. 1.5 ± 0.1 ms, *p* = 0.63), AHP amplitude (-17.6 ± 1.6 vs. -14.9 ± 1.6 mV, *p* = 0.25), and AHP half width (28.9 ± 6.9 vs. 32.7 ± 6.0 ms, *p* = 0.11 Mann–Whitney *U* test), between groups. These data suggest the major conductances that shape AP properties in PVINs are not altered during aging.

### Excitatory Input to Parvalbumin Expressing Interneurons

**FIGURE 2:**
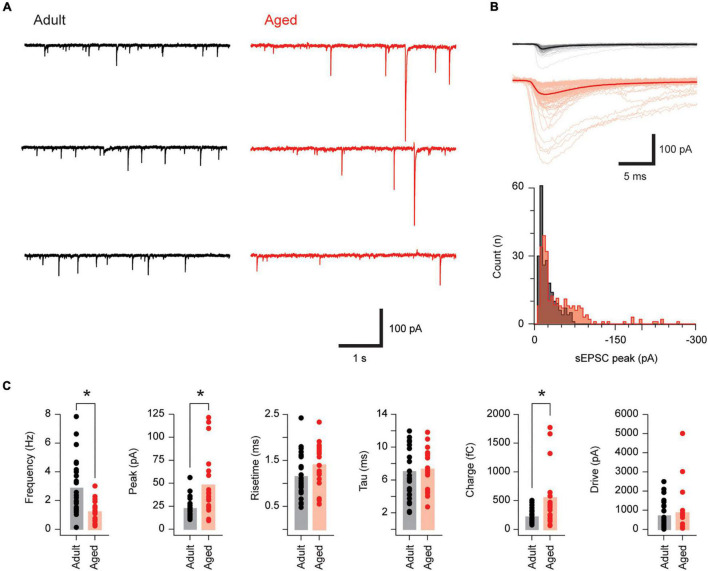
Excitatory synaptic drive is unchanged in aged PVINs. **(A)** Traces show continuous sEPSC recordings from adult and aged PVINs. Note, sEPSC amplitude is clearly greater in aged PVINs. **(B)** Upper overlaid sEPSC traces are captured from the data in **(A)**. The average of these captured events (heavy trace) emphasizes the increased sEPSC amplitude in aged animals. Lower plots show amplitude histograms highlighting a population of right shifted larger sEPSCs in the aged recording. **(C)** Plots comparing group data for sEPSC properties in PVINs from adult and aged mice. In aged mice, sEPSC amplitude and charge are increased whereas sEPSC frequency is reduced. sEPSC time course (rise and decay time) and synaptic drive (charge per second) are unchanged in aged PVINs (asterisks indicate significant differences, *p* < 0.05). Each data point represents the mean of at least 20 events.

We next assessed the impact of advanced age on the excitatory drive that recruits PVINs by recording sEPSCs in adult and aged neurons (*n* = 26 and 19, respectively; Figure 2). sEPSC amplitude was markedly increased in aged PVINs (22.3 ± 1.9 vs. 47.5 ± 7.9 pA, *p* < 0.001 Mann–Whitney *U* test, Figures 2A,B), whereas sEPSC frequency was more than halved in aged PVINs (2.85 ± 0.37 vs. 1.18 ± 0.18 Hz, *p* < 0.001 Mann–Whitney *U* test). In contrast, sEPSC rise time (1.14 ± 0.09 vs. 1.39 ± 0.11 ms, *p* = 0.083) and decay time constant (6.99 ± 0.64 vs. 7.27 ± 0.59 pA, *p* = 0.76) were similar in the two groups (Figure 2C). Together, these values led to an increased sEPSC charge (544 ± 114 vs. 214 ± 24 fC, *p* = 0.01 Mann–Whitney *U* test), however, excitatory drive, calculated as sEPSC charge per second, did not differ in adult and aged PVINs (844 ± 290 vs. 698 ± 136 pA, *p* = 0.65 Mann–Whitney *U* test). Thus, the overall effect of increased sEPSC amplitude and decreased sEPSC frequency suggest excitatory synaptic drive to PVINs remains constant with age.

### Inhibitory Input to Parvalbumin Expressing Interneurons

**FIGURE 3:**
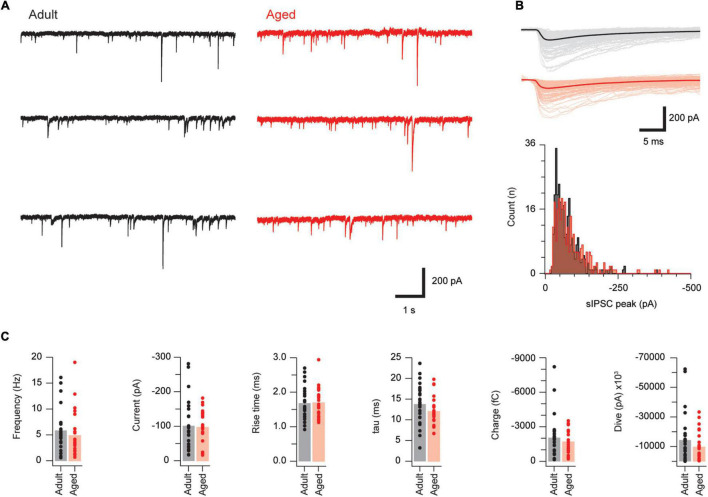
Synaptic inhibition is similar in adult and aged PVINs. **(A)** Representative traces showing sIPSCs in adult (left, black) and aged (right, red) PVINs. **(B)** Upper overlaid sIPSC traces captured from records in **(A)** with averaged responses indicated by heavier traces. Lower plots show amplitude histograms highlighting similar amplitude distributions in adult and aged sIPSC recordings. **(C)** Plots showing group data for sIPSC properties. Note, the similarly in all sIPSC properties measured in adult and aged mice. Each data point represents the mean of at least 20 events.

We next examined inhibitory drive to PVINs by recording sPSCs in adult and aged mice (*n* = 27 and 24, respectively; Figure 3). The inhibitory nature of sPSCs was confirmed using pharmacology, as the use of a CsCl-based internal solution in these experiments, and associated high intracellular Cl^–^ concentration, meant that both inhibitory and excitatory synaptic inputs present as inward currents. Recordings were examined under baseline conditions and following bath application of GABA, glycinergic, and glutamatergic antagonists. This showed that bicuculline and strychnine completely blocked sIPSCs (2.71 ± 0.88 vs. 0.02 ± 0.01 Hz, *p* = 0.004 Wilcoxon matched-pairs signed rank test, *n* = 9). In contrast, CNQX (*n* = 10) reduced sIPSC frequency (3.01 ± 0.68 vs. 2.16 ± 0.43 Hz, *p* = 0.047 paired *T*-Test), but had no effect on amplitude (168.2 ± 37.0 vs. 177.8 ± 40.3 pA, *p* = 0.38 Wilcoxon matched-pairs signed rank test), or decay time constant (15.41 ± 1.42 vs. 15.76 ± 0.76 pA, *p* = 0.77 Paired *T*-Test). This pharmacology is consistent with sIPSC recordings. Average sIPSC amplitude (101.3 ± 13.7 vs. 97.3 ± 10.1 pA, *p* = 0.72 Mann–Whitney *U* test), rise time (1.68 ± 0.09 vs. 1.70 ± 0.09 ms, *p* = 0.92 Mann–Whitney *U* test) decay time constant (13.79 ± 0.91 vs. 12.05 ± 0.74 pA, *p* = 0.15) and sIPSC frequency (5.85 ± 0.76 vs. 4.89 ± 0.90 Hz, *p* = 0.12, respectively) did not change with age. This was also the case for the derived sIPSC properties synaptic charge and synaptic drive (1702 ± 218 vs. 2045 ± 348 fC, *p* = 0.41 Mann–Whitney *U* test; and 9,583 ± 2,048 vs. 14,281 ± 3,177 pA, *p* = 0.22 Mann–Whitney *U* test, Figure 3C). These data suggest inhibitory control of PVINs remains stable with advancing age.

### Inhibitory Connectivity of Parvalbumin Expressing Interneurons With Other Dorsal Horn Neurons

**FIGURE 4:**
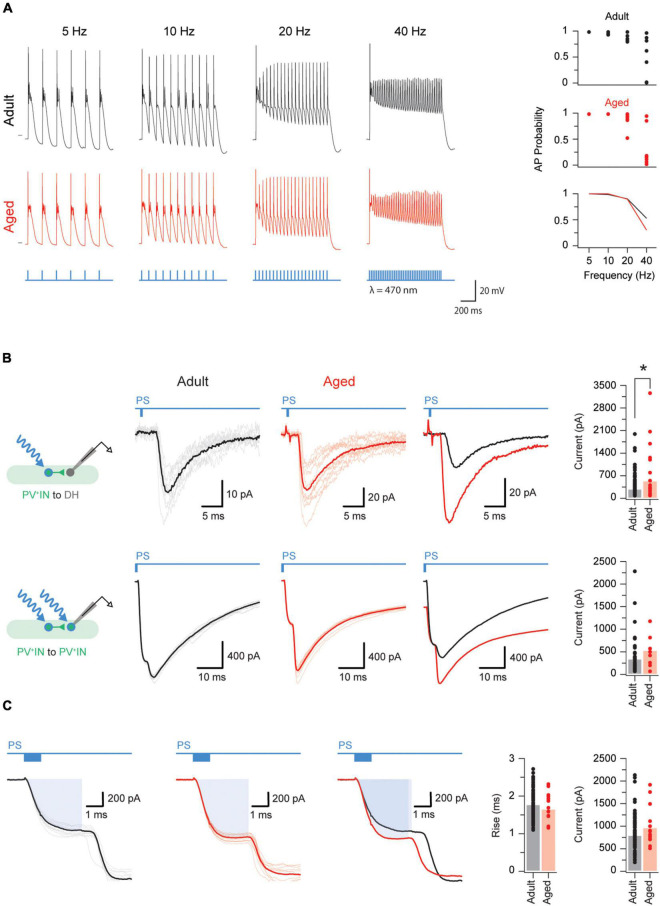
Aged PVINs increase their postsynaptic output. **(A)** Traces showing photostimulation-evoked AP discharge in adult and aged PVINs at increasing stimulation frequencies. Photostimulation pulses (1 ms duration) and stimulation protocol are shown in blue. The short horizontal black bars on the 5 Hz discharge traces indicate a membrane potential of −60 mV. Top two plots on the right summarize the reliability of evoked AP discharge at the four photostimulation frequencies for adult and aged PVINs. Bottom graph compares the mean probability for AP discharge at different photostimulation frequencies. **(B)** Voltage clamp recordings (−70 mV holding potential) showing PVIN photostimulation-evoked inhibitory postsynaptic currents (oIPSCs) generated in unidentified neurons (top traces) and PVINs (bottom traces). Schematics to the left show each photostimulation and recording configuration. Left traces are recorded from adult mice, and middle traces from aged mice (10 consecutive sweeps and average overlayed in each). Right traces show overlaid averages. Note, oIPSC responses are markedly larger at PVIN to unidentified cell connections in aged animals. Group data plots (far right) compare oIPSC amplitude for each recording configuration and show that oIPSC amplitude is increased at PVIN to unidentified cell connections in aged mice. **(C)** Voltage clamp recordings (−70 mV holding potential) from adult (left) and aged (middle) PVINs on an expanded time scale to show the photocurrent phase of the photostimulation response (highlighted by blue rectangle). Average overlaid photocurrent responses (right trace) show they are similar in recordings from adult and aged animals. Group data (far right plots) compare photocurrent rise time and amplitude in adult and aged PVINs. The similarity in these two measures suggest age related changes in photocurrent properties does not explain the larger oIPSCs observed at PVIN to unidentified cell connections in aged mice. Each data point represents the mean of 10 consecutive traces events. *p < 0.05.

We next examined the properties of the inhibitory connections PVINs make with DH neurons in adult and aged mice (Figure 4). We did this because our previous work has shown inhibitory PVIN connections within DH circuits are important for restricting the influence of small- and large-fiber peripheral inputs to superficial and deep layers of the DH, respectively, (Hughes et al., 2012). We made two types of recording: (1) from randomly selected ChR2/YFP negative neurons (hereafter termed “unidentified neurons”); and (2) and from PVINs. All recordings were restricted to cells located within the PV positive neuron plexus (LIIi-III) to ensure photostimulation maximally activated the PVIN population.

We first tested the capacity of adult and aged PVINs to follow increasing photostimulation frequencies (5–40 Hz, Figure 4A). Based on their AP discharge, all adult and aged PVINs could faithfully follow photostimulation at frequencies up to 10 Hz (Figure 4A, plots on right). Some neurons, however, in both adult and aged PVINs, could follow photostimulation frequencies up to 40 Hz.

Photostimulation elicited oPSCs in both unidentified and PVINs. Example voltage clamp recordings for PVIN to an unidentified cell and PVIN to PVIN connections are shown in Figure 4B. Recordings from both types of connections contained short latency (3.9 ± 0.1 ms and 4.8 ± 0.2 ms for unidentified and PVINs, respectively) and limited jitter responses (0.6 ± 0.1 ms and 0.4 ± 0.1 ms for unidentified and PVINs, respectively). Such short latency and jitter values are consistent with the existence of monosynaptic connections (Lu and Perl, 2003). Using these criteria, 77% (104/136) of the PVIN to unidentified cell connections and 56% (48/85) of the PVIN-PVIN connections in adult mice are monosynaptic. In aged mice, however, monosynaptic oPSC were observed in almost all PVIN-unidentified cell connections (96%; 25/26 cells), but were unchanged at PVIN-PVIN connections (53%; 8/15). As well as changes in connectivity, the amplitude (or strength) of the connections between PVINs and unidentified cells was ∼2 fold larger in aged vs. adult mice (516.9 ± 157.5 vs. 271.5 ± 31.8 pA, *p* = 0.016 Mann–Whitney *U* test, Figure 4B). In line with a minimal contribution of excitatory PVINs in the region sampled (Gradwell et al., 2021), and our above results recording spontaneous inhibitory currents using a CsCl loaded internal solution, oPSCs were completely abolished by application of bicuculine and strychnine (5.3 ± 1.1 pA, *p* < 0.001 Wilcoxon matched-pairs signed rank test), but insensitive to CNQX (305.1 ± 66.5 vs. 273.4 ± 63.0 pA, *p* = 0.853 Wilcoxon matched-pairs signed rank test).

To assess if the size of the photocurrent generated in adult vs. aged PVINs influenced the above results we compared photocurrent properties (Figure 4C). Both rise time (1.76 ± 0.04 vs. 1.63 ± 0.10 ms, *p* = 0.25) and amplitude (787 ± 42 vs. 952 ± 110 pA, *p* = 0.11) of optically evoked photocurrents were similar in adult and aged recordings (Figure 4C, right plots), confirming expression or properties of ChR2 in adult vs. aged PVINs did not influence our results. Together this analysis suggests PVINs: (1) are more strongly connected to unidentified neurons in both adult and aged mice (Chi square test, *p* < 0.01); and (2) their connectivity with unidentified INs increases with age (Chi square test, *p* < 0.01).

As fast inhibitory postsynaptic currents in the DH are generated by the release of GABA and glycine (or both), we further examined the optically generated IPSCs to determine whether the contribution of GABAergic and glycinergic transmission changed with age. We first fit the decay phase of monosynaptic oPSCs with a single exponential and observed slower decay time constants in aged recordings (21.66 ± 66 vs. 34.86 ± 6.88 ms). As PVINs are known to release both GABA and glycine, we also fit the decay phase of monosynaptic oPSCs with a double exponential. This approach is based on the markedly differing kinetics of GABAergic and glycinergic currents in mouse DH neurons (Graham et al., 2003; Tadros et al., 2014), exhibiting slow and fast decay times, respectively. A substantial improvement in the “goodness of fit” between single and double exponential fitting indicated both GABAergic (slow) and glycinergic (fast) components existed in the oIPSC. The decay of oIPSCs in adult mice were evenly split between single (13/22: 59%) and double (9/22: 41%) exponential fits. In contrast, the decay phase of most oPSCs in aged mice were best fit by a double exponential (7/9: 78%). These differences in decay time course were statistically significant (Chi square statistic comparing incidence of double fits to IPSC decay = 0.026). Together this suggests there is an increased contribution of GABAergic inhibitory transmission at PVIN synapses on other DH populations in aged animals.

## Discussion

The impetus for this study grew out of the growing appreciation that pain increases with age and the changes might be explained by an increased level of excitability in neurons within the DH. Evidence for increased excitability during aging has been studied at the first central node of the pain neuroaxis, but to our knowledge has not been examined in a specific neuronal population. We choose PVINs as they are important for pain behavior in the DH (Hughes et al., 2012; Petitjean et al., 2015). Our study has three major findings. First, the overall excitability of PVINs increases with age. Second, excitatory and inhibitory drive to PVINs does not change with age, although there are some changes in the components that contribute to excitatory drive (i.e., larger but less frequent sEPSCs in aged mice). Finally, the inhibitory connections PVINs make with unidentified DH neurons strengthens with age and there is a shift from mixed (glycinergic and GABAergic) to GABAergic inhibitory transmission in aged animals.

Before the above results can be interpreted in terms of pain circuits and behaviors it is crucial to appreciate that the ultimate effect of the above changes depend on the precise phenotype of the recorded neuron. Recent work from our group has shown that the PVINs are split 50:50 into excitatory and inhibitory phenotypes (Gradwell et al., 2021). This complicates interpretation of our aging data for PVINs, but is helped somewhat by our reasonably advanced understanding of the properties of inhibitory and excitatory PVINs in spinal circuits. Specifically, excitatory PVINs have small somas and dendritic arbors and provide weak excitation to INs and projection neurons (PNs) in laminae I and IIo. These properties are consistent with a role in the recruitment of pain circuits. In contrast, inhibitory PVINs, are large islet cells, provide GABAergic presynaptic inhibition to myelinated low-threshold mechanoreceptor afferent terminals in laminae IIi as well as mixed post synaptic inhibition (GABAergic and glycinergic) to neurons in the DH. These properties are consistent with inhibitory PVINs playing a role in segregating innocuous tactile input from pain-processing circuits (Zeilhofer et al., 2012; Petitjean et al., 2015).

### Altered Intrinsic Excitability in Parvalbumin Expressing Interneurons

Intrinsic excitability governs the responsiveness of any neuron to its synaptic inputs (Llinas, 1988; Marder et al., 1996). Importantly, it is now well accepted that intrinsic excitability is not static but can be modified by changes in: ion channel expression and density (Balachandar and Prescott, 2018); underlying tonic synaptic currents (Gradwell et al., 2017); neuromodulator actions (Nadim and Bucher, 2014); and excitatory and inhibitory synaptic inputs (Lambo and Turrigiano, 2013; Tadros et al., 2014). In this study we showed that the excitability in a well-understood type of DH IN, PVINs, also changes with age. Specifically, AP discharge shifts toward the TF profile and AP discharge duration increases (Figure 1). This increased excitability matches our recent work comparing unidentified superficial DH neurons (LI-II) in adult and aged mice (Mayhew et al., 2019), however, that study examined excitability in four vs. the two discharge types that dominate in PVINs. Broadly speaking the increased excitability of PVINs would lead to an enhanced AP output in response to a given synaptic drive in the aged animals. The consequences of any increased output, however, would depend on the neurochemical phenotype of the affected cell and its role in circuit function. This is a difficult question to tackle in a mixed population of neurons, however, we have gained some insight by using optogenetic activation of PVINs to study the consequences of the increased output of the inhibitory PVIN population (see heading: *Role of inhibitory PVINs in aged DH circuits*). Future studies using intersectional approaches that selectively label excitatory and inhibitory PVINs will be required to further resolve these aging effects in each PVIN subpopulations (Das Gupta et al., 2021).

The mechanisms underlying the increased excitability in aged PVINs is not clear, however, we know something about the role of conductances that shape AP discharge in DH neurons. For example, the TF discharge profile is favored by neurons where Na^+^ and delayed rectifier K^+^ (K_*DR*_) current expression is high, and the density of A-type K^+^ currents is low (Melnick et al., 2004b; Balachandar and Prescott, 2018). IB discharge has been shown to be favored when Na^+^ currents are reduced relative to K_*DR*_ (Melnick et al., 2004a), whereas a high density of A-type K+ currents produce distinct DF discharge (Smith et al., 2015). Thus, alteration of just three conductance’s has the capacity to dramatically alter AP discharge. In fact, substantial work has highlighted how the combined sum of inward and outward voltage sensitive conductance’s explains the breadth of AP discharge throughout the nervous system (Prescott et al., 2008). Further, distinct channel expression patterns or differences in the ratio (or levels) of a similar series of channels can reproduce AP discharge diversity, while also predicting the role of stimulus intensity and membrane potential in the ability of DH populations to switch patterns of AP discharge (Balachandar and Prescott, 2018). Considering this literature, future work to quantify and compare key conductance’s in young and aged DH populations, or quantifying changes in expression levels using modern techniques such as single cell RNA-Seq, will be required to determine the mechanisms that increase TF discharge in aged PVINs.

### Synaptic Inputs to Parvalbumin Expressing Interneurons

Overall, excitatory drive to PVINs was not altered with age (Figure 2). This contrasts with our previous work where we showed excitatory drive to unidentified DH neurons decreased with age (Mayhew et al., 2019). There are several potential explanations for this discrepancy. First the Mayhew study targeted neurons in the superficial DH (i.e., laminae I-II) whereas our recordings from PVINs targetted deeper layers (laminae IIi-III). Second as ∼70% of neurons in the DH are thought to be excitatory (Polgar et al., 2013) the majority of recordings in the Mayhew study were likely from excitatory interneurons, contrasting the approximately 50/50 split that we have recently reported for PVINs in PV*^cre^*;Ai32 mice (Gradwell et al., 2021). These sampling difference likely contribute to the contrasting findings of both studies and suggests age-related changes to excitatory signaling are not uniform throughout the DH. Instead, altered excitatory drive is likely to be region, circuit, and neuron specific.

There were, however, some very notable changes in sEPSC amplitude and frequency in aged PVINs (Figure 2). Specifically, aging was accompanied by a two-fold increase in sEPSC amplitude and equally significant decrease in sEPSC frequency. Assessment of amplitude distributions (see Figure 2B) highlighted an increase in larger sEPSC amplitudes to the right of the distributions, along with occasional large events contribution to this finding. It is important to note that as these are spontaneous EPSC recordings, including action potential dependent and independent events, an increase in spontaneous AP discharge may contribute to larger events in aged PVINs. Alternatively, larger sEPSCs could be explained by increases in postsynaptic receptor density, the number of excitatory synapses on PVINs, or excitatory synapses located closer to soma in PVINs (Bekkers, 1994; Graham et al., 2006). Recordings of miniature excitatory postsynaptic currents (mIPSCs) would be required to differentiate these potential mechanisms. Alternatively, reduced sEPSC frequency in aged PVINs could arise from reduced spontaneous AP discharge in aged spinal cord slices or be shaped by a reduction in the number of excitatory synapses onto PVINs, or lowered neurotransmitter release probability in presynaptic glutamatergic neurons (Turrigiano, 1999). mEPSC recordings, morphological and immunohistochemical examination of aged PVINs would provide further insight into mechanisms underlying larger but infrequent sEPSCs observed in the present study.

We observed no change in inhibitory drive to PVINs and there were no notable changes in sIPSC amplitude or frequency with age. This matches our previous findings on unidentified DH neurons in the mouse SDH (Mayhew et al., 2019) and suggests activity in inhibitory interneurons remains relatively stable during aging. Work on the impact of aging on inhibitory drive has been undertaken within other CNS regions. Increased inhibition has been reported within the prefrontal cortex (Bories et al., 2013; Banuelos et al., 2014), whereas reduced inhibition has been reported in parietal cortex (Schmidt et al., 2010), somatosensory cortex (David-Jurgens and Dinse, 2010), primary motor cortex (Heise et al., 2013), and hippocampus (Potier et al., 2006; McQuail et al., 2015). These data suggest the effect of aging on inhibitory drive is circuit and region specific.

### Role of Inhibitory Parvalbumin Expressing Interneurons in Aged Dorsal Horn Circuits

Using optogenetics and a chloride-based internal in our recording pipettes we were able to study the functional output of inhibitory PVINs. Notably, we could study PVIN connections with unidentified neurons and between PVINs. These experiments showed the strength of inhibitory synaptic connections (based on oIPSC amplitude, latency and jitter values) between PVINs and unidentified DH neurons increased with age. This was not the case for PVIN-PVIN connections. In broad terms this means any changes in connectivity between cell types that accompanies aging is not uniform in DH. Reconciling this main finding with other work on inhibitory connections in the aged CNS and DH is difficult as most work, including our previous study in the DH (Mayhew et al., 2019), simply measure spontaneous or miniature IPSCs. Spontaneous IPSC recordings represent the average properties of all inhibitory inputs to a given neuron, regardless of origin. In contrast, the oPSCs we recorded represent a synaptic current that is driven by AP discharge in several PVINs. Regardless of these complexities our data clearly show that the strength of PVIN-mediated inhibition is enhanced in unidentified DH neurons. These data are in line with the enhanced GABAergic dominance of sIPSCs observed in our previous work on unidentified neurons in the superficial DH of aged animals (Mayhew et al., 2019). Thus, a shift to GABAergic inhibitory signaling seems to occur in both superficial and deeper layers of the DH during aging. Notably, this age-related change may be greater in deeper vs. superficial laminae of the DH as GABAergic and glycinergic signaling dominate superficial and deep layers, respectively, in naïve adult rodents (Cronin et al., 2004; Anderson et al., 2009).

Another important determinant of inhibitory synaptic strength, not assessed in our data, is the reversal potential of chloride. This critical factor sets the driving force for chloride conductance’s in adult CNS neurons through expression of the K^+^-Cl^–^ co-transporter KCC2. Relevant to the current study, KCC2 is developmentally regulated (Watanabe and Fukuda, 2015), regionally distinct in the DH (Ferrini et al., 2020), and has been shown to vary in neuropathic pain states (Coull et al., 2003). Our use of a CsCl-internal solution, and associated loading of intracellular chloride, precluded an assessment of chloride reversal. Thus, work with appropriate recording conditions will be required to assess if aged DH circuits also undergo a shift in the electrochemical gradient of chloride conductance’s. Our data demonstrating other changes to inhibitory synaptic function suggest this as a worthwhile future direction.

As for the differences in temporal properties of glycinergic and GABAergic currents, the above changes are likely to impact the temporal fidelity of PVIN-mediated inhibition. Enhanced GABAergic transmission would increase the potential for summation of multiple inhibitory inputs while reducing precision. Just how such changes would ultimately impact sensory processing in the DH cannot be assessed in the experiments described here. However, we can speculate based on our knowledge of the role PVINs play in pain processing. For example, our finding that GABAergic signaling from PVINs is enhanced may also have implications for the axoaxonic (presynaptic) synapses these cells make onto low-threshold mechanoreceptors (Hughes et al., 2012). If axoaxonic synapses also strengthen with age, as shown above for axodendritic synapses, PVIN activity would likely promote more powerful presynaptic control of the low-threshold mechanoreceptor afferent population and reduce allodynia. Further insight into the effects of enhanced GABAergic signaling at the systems level of analysis could be obtained by comparing pain behavior during *in vivo* optogenetic activation of PVINs in young and aged animals (Smith et al., 2015).

In conclusion, PVINs have been shown to act as “gatekeepers” for touch evoked pain (Petitjean et al., 2015). Thus, their increased capacity for sustained repetitive discharge and potential enhancement of their presynaptic inhibitory function would maintain the capacity of PVINs to prevent touch induced pain with advancing age. The stimulus that drives these age-related changes in PVIN function is unclear, however, it is now well established that the aged CNS exists in a chronic neuroinflammatory state (Simen et al., 2011; Abdel-Aziz et al., 2014; Mayhew et al., 2020). Thus, any change to PVIN function may simply be a compensatory mechanism to stabilize spinal sensory processing and maintain the segregation of tactile and nociceptive circuits. In the context of the current study, using naïve uninjured animals, this compensation may be effective at reducing age-related pain. However, after injury the capacity of PVINs to maintain normal sensory processing in aged animals may be overwhelmed or compromised. Future work on PVINs using various murine pain models at differing ages will be required to explore this hypothesis.

## Data Availability Statement

The original contributions presented in this study are included in the article/supplementary material, further inquiries can be directed to the corresponding author.

## Ethics Statement

The animal study was reviewed and approved by the University of Newcastle Animal Care and Ethics Committee.

## Author Contributions

MG, KS, CD, DS, RC, DH, and BG conceived and designed the research study. MG conducted experiments and acquired data. MG, KS, and BG analyzed data. MG, RC, and BG wrote the manuscript. All authors edited the final version of the manuscript.

## Conflict of Interest

The authors declare that the research was conducted in the absence of any commercial or financial relationships that could be construed as a potential conflict of interest.

## Publisher’s Note

All claims expressed in this article are solely those of the authors and do not necessarily represent those of their affiliated organizations, or those of the publisher, the editors and the reviewers. Any product that may be evaluated in this article, or claim that may be made by its manufacturer, is not guaranteed or endorsed by the publisher.
